# A Novel Bioreactor for Reconstitution of the Epithelium and Submucosal Glands in Decellularized Ferret Tracheas

**DOI:** 10.3390/cells11061027

**Published:** 2022-03-18

**Authors:** Albert C. Pai, Thomas J. Lynch, Bethany A. Ahlers, Vitaly Ievlev, John F. Engelhardt, Kalpaj R. Parekh

**Affiliations:** 1 Department of Cardiothoracic Surgery, University of Iowa Hospitals and Clinics, Iowa City, IA 52242, USA; albert-pai@uiowa.edu; 2 Department of Anatomy and Cell Biology, University of Iowa, Iowa City, IA 52242, USA; thomas-lynch@uiowa.edu (T.J.L.); bethany-ahlers@uiowa.edu (B.A.A.); vitaly-ievlev@uiowa.edu (V.I.); john-engelhardt@uiowa.edu (J.F.E.)

**Keywords:** bioreactor, tracheal transplant, airway submucosal gland, airway stem cells, ferret

## Abstract

Tracheal grafts introduce the possibility to treat airway pathologies that require resection. While there has been success with engraftment of the surface airway epithelium (SAE) onto decellularized tracheas, there has been minimal advancement in regenerating the submucosal glands (SMGs). We designed a cost-effective open-system perfusion bioreactor to investigate the engraftment potential of ferret SAEs and murine myoepithelial cells (MECs) on a partly decellularized ferret trachea with the goal of creating a fully functional tracheal replacement. An air–liquid interface was also arranged by perfusing humidified air through the lumen of a recellularized conduit to induce differentiation. Our versatile bioreactor design was shown to support the successful partial decellularization and recellularization of ferret tracheas. The decellularized grafts maintained biomechanical integrity and chondrocyte viability, consistent with other publications. The scaffolds supported SAE basal cell engraftment, and early differentiation was observed once an air–liquid interface had been established. Lastly, MEC engraftment was sustained, with evidence of diffuse SMG reconstitution. This model will help shed light on SMG regeneration and basal cell differentiation in vitro for the development of fully functional tracheal grafts before transplantation.

## 1. Introduction

Tracheal resection of neoplasms or stenotic lesions may result in long-segment defects. Generation of tracheal defects >50% of the total length of trachea in adults, or >30% in pediatric patients, risks the dehiscence of primary anastomoses due to excessive tension on the repair. As such, there is a clinical need for tracheal replacement, and several conduits have been explored to bridge the gap, including autologous tissue composites, aortic allografts, solid prostheses, tracheal transplantation, and bioengineered tracheas [1,2]. Within this latter category, decellularization and recellularization of grafts has emerged as a popular method for studying stem cell implantation; however, the ideal candidate for generating a fully functional tracheal graft has yet to be elucidated. Furthermore, variations in bioreactor design and the source of cadaveric tracheas can financially limit the throughput and quality of experiments. Here, we designed a cost-effective bioreactor capable of decellularizing and recellularizing a ferret trachea and investigated the engraftment potential of myoepithelial cells (MECs) and surface airway basal cells to create a wholly functional organ.

Decellularized grafts are an appealing transplant option, given their implied absence of mismatched major histocompatibility complexes (MHCs) and the theoretically lower incidence of recipient-derived rejection [3,4]. The remaining micro- and macroarchitecture and extracellular matrices are then capable of supporting reseeded cells for organ function [5,6]. Methods of decellularizing tracheas include mechanical means (e.g., freeze–thaw) [7], enzymatic treatments [8], and detergent protocols with equivocal superiority [9,10,11,12,13]. Regardless of the method, the negative consequences of using acellular scaffolds have been realized, as decellularized tracheal scaffolds in vivo have been associated with postoperative stenoses due to compromised cartilage [14,15]. At this time, cartilage regeneration remains a challenge in decellularized grafts.

Aoki et al. recently addressed this issue with a partly decellularized porcine trachea [16]. Through the use of a custom-built closed-system perfusion bioreactor, a detergent-based protocol de-epithelialized the lumen while preserving a majority of the chondrocytes and the biomechanical integrity. There is evidence to suggest that tracheal chondrocytes are immune-privileged and can forgo the decellularization process [17]. The graft was then reseeded with expanded airway stem cells to create a chimeric allograft. While remnants of the airway submucosal glands (SMGs) were notably present, prior studies have emphasized that the surface airway epithelium (SAE) is the primary target of allograft rejection [18,19], and successful eradication of the luminal cells reduces the bulk of the antigen burden which lessens the risk of rejection. Subsequent re-epithelialization of denuded surfaces has also been demonstrated to be vital in staving off obliterative lesions in implanted tracheas [20,21].

Presence of SMGs within tracheal implants may also enhance grafts’ functionality, since these structures harbor stem cells for the SAE and secrete factors that are important in innate immunity of the trachea. However, SMGs harbor MHC proteins which stimulate CD4+ T cell proliferation within transplanted grafts [22], and thus the optimal tracheal graft should ideally decellularize the SMGs as well. The tradeoff of doing so would be an increased risk of bacterial infection, which was demonstrated in heterotopically transplanted tracheas without SMGs [23]. With that in mind, a clinically optimal tracheal graft would be one with reconstructed SMGs expressing self-antigens that retain immune function and repair capability. A fascinating contribution towards the development of such an airway was the de novo generation of SMGs conducted in Matrigel studies by Wu et al. using human nasal basal cells [24]. In these studies, basal cells cultured for a period of over 40 days with high levels of epidermal growth factor (EGF) were capable of generating tubuloacinar cells expressing lysozyme, mucin (MUC5B), peripheral keratin 14 (KRT14), and α-smooth muscle actin. These peripheral cells were reminiscent of SMG-associated myoepithelial cells (MECs). Another set of recent lineage-tracing experiments elucidating the multipotent nature of MECs have identified these cells as an alternative candidate for a role in SMG regeneration [25,26]. After severe injury, MECs adopt a basal cell phenotype to regenerate SAE, establish lasting progenitors, and contribute to the repair of damaged SMG structures [25,26].

Orthotopic transplantation of these partly decellularized grafts in an animal model is undeniably the ultimate intent. While pig airways are anatomically comparable with humans in size, the maintenance of large animals can be costly, thereby limiting the effective size of a study. Fortunately, ferrets (*Mustela putorius furo*) have been historically recognized as an animal model for the study of infectious respiratory illnesses, cystic fibrosis (CF) [27,28,29], and chronic lung allograft dysfunction (CLAD) after lung transplantation [30,31]. Ferrets uniquely have a histologic profile of chronic rejection identical to that of humans, and their use may serve a similar purpose in recognizing tracheal graft rejection. Their generously long tracheas (~90 mm) relative to their small body size allows for a substantial amount of useable tracheal tissue and for manageable monetary upkeep [32]. Additionally, the recent development of a transgenic fluorescent ferret also offers a unique opportunity to track the colonization, migration, and retention of cells after reseeding onto a decellularized graft [33]. These are compelling reasons for selecting ferrets as an alternative model for tracheal transplant studies.

In this study, we addressed the issues above by creating a cost-effective bioreactor capable of luminally decellularizing and re-epithelializing a ferret trachea. We investigated the re-epithelialization and SMG generation potential of two cells lines: primary tracheal airway basal cells and tracheal myoepithelial cells from a transgenic ROSA26-CAG-^LoxP^ tdTomato^StopLoxP^ EGFP (ROSA-TG) ferret and mouse, respectively.

## 2. Materials and Methods

### 2.1. Animals

All animal experimentation was performed in accordance with protocols approved by the Institutional Animal Care and Use Committees of the University of Iowa.

Mice: Mice of the Cre-driver strain of Tg(Acta2-cre/ERT2)51Pcn(*ACTA2-Cre^ERT2^*) were bred as described [34] and purchased from Jackson Laboratory (Bar Harbor, ME, USA). Cre-mediated recombination was induced using a single intraperitoneal injection of tamoxifen (2 mg/kg for adults).

Ferrets: A dual-fluorescent Cre-reporter ferret was engineered by CRISPR/Cas9-mediated homology-independent insertion as described [31]. Wild-type female ferrets were obtained from Marshall Farms (Rose, NY, USA).

### 2.2. Tissue Processing and Cell Isolation

Epithelia from resected mouse and ferret tracheas were isolated using a sequential enzymatic protocol with slight modifications as previously described [25]. In brief, animals were euthanized with an overdose of Euthasol^®^ (pentobarbital sodium and phenytoin sodium) (Virbach AH, Inc., Prod# 710101, Fort Worth, TX, USA) and then topically cleaned with 70% ethanol. Cervical tracheas were collected sterilely and decontaminated for 24 h in a solution of Minimum Essential Media (MEM) (Gibco, St. Louis, MO, USA), imipenem/cilastatin (50 µg/mL), ceftazidime (50 µg/mL), amphotericin B (125 µg/mL), gentamicin (50 µg/mL), and 1% penicillin/streptomycin at 4 °C. Ferret surface airway epithelia (SAE) were enzymatically isolated by digesting tracheal tissue in 5 mg/mL of Pronase (Roche, Cat#10165921001, Mannheim, Germany) in F12 media for 60 min. Cells were then pelleted and cultured on 804G-conditioned plates with modified small airway growth media (SAGM) (Lonza, Cat#CC-3118, Basel, Switzerland) with the addition of 10 μM Y-27632 (Tocris, Cat#1254, Minneapolis, MN, USA), 1 μM DMH-1 (Tocris, Cat#4126, Minneapolis, MN, USA), 1 μM A83-01 (Tocris, Cat#2939, Minneapolis, MN, USA), and 1 μM CHIR 99,021 (Tocris, Cat#4423, Minneapolis, MN, USA) for mouse or with Pneumacult Ex-Plus (Stemcell Technologies, Cat#05008, Vancouver, BC, Canada) for ferrets.

Myoepithelial cells (MECs) of mice were isolated from submucosal glands. The tracheas were similarly digested in 3 mg/mL of Pronase, and the residual tissue was further digested in a 1:1 mixture of 10X collagenase/hyaluronidase (Stemcell Technologies, Cat#07912, Vancouver, BC, Canada) in F12 media (ThermoFisher Scientific, Cat#11765054, Waltham, MA) for 45 min and supplemented with 0.02% EDTA/0.05% trypsin solution (Life Technologies, CA#25300054, Carlsbad, CA, USA). Pelleted cells were expanded with modified SAGM (as above). After sufficient expansion, cells were sorted by fluorescence in flow cytometry to achieve a pure GFP-positive population, representing *ACTA2*-lineage cells.

### 2.3. Bioreactor Design

**Figure 1:**
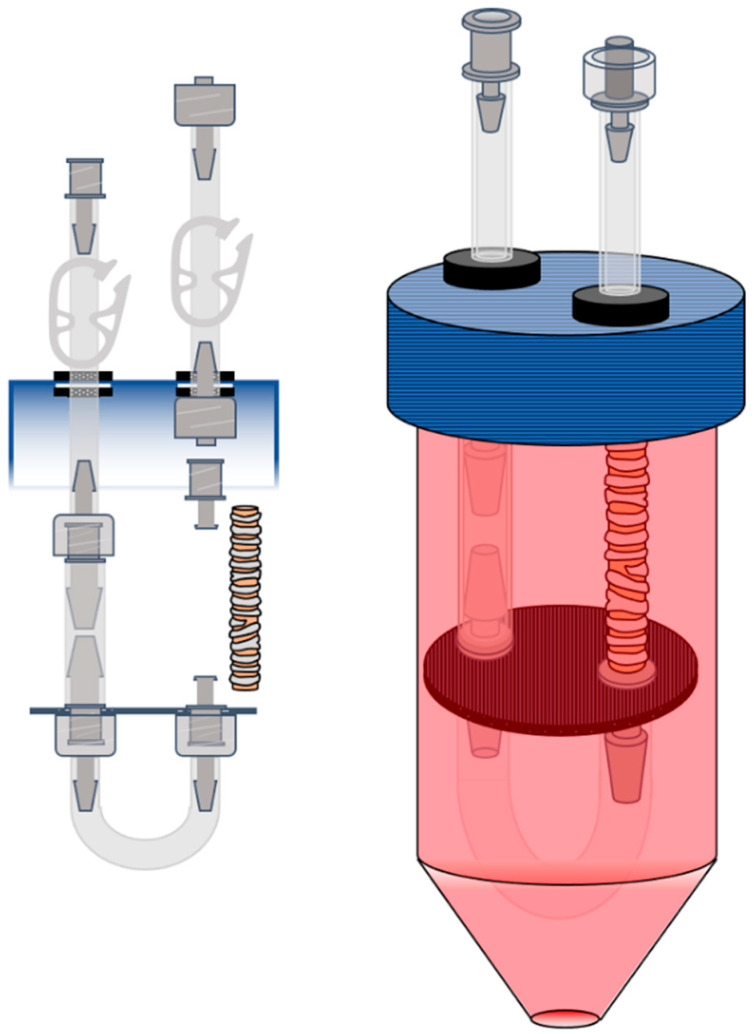
Bioreactor setup. A 50 mL conical tube cap was modified to accommodate two rubber grommets (11/32″ [8.73 mm] OD × 1/8″ [3.175 mm] ID). Silastic tubing and Luer adapters were connected as described in the article to fashion a “J” loop. A 5-cm (if decellularizing) or 3-cm (if recellularizing) length of ferret trachea was fixed to the cuffs with 2-O silk. The entire circuit was then submerged in 40 mL of basic media consisting of F12:DMEM and 10% FBS. The outer media was exchanged every 48 h.

The bioreactor was inspired by Loy et al. [35] and was designed for both decellularization and recellularization. A 50-mL conical tube cap was fashioned as depicted in Figure 1 by drilling two holes with a ¼″ drill bit (6.35 mm). Two rubber grommets (Hillman, 11/32″ [8.73 mm] outer diameter × 1/8″ [3.175 mm] inner diameter, Tempe, AZ, USA) were fitted into the holes to create a hermetic seal. A rubber O-ring (Uxcell, Kwai Fong New Territories, China) with a 6-mm inner diameter was placed around the inner diameter from the underside of the cap to reinforce the seal. A 5-cm segment of 2 × 1 mm Silastic tubing (BaoDing ShenChen Precision Pump, BaoDing City, China) was threaded through each grommet, and 1/8″ (3.175 mm) male Luer ends were connected from the underside of the cap, and the 1/8″ (3.175 mm) male and female Luer ends were connected to the external ends of the Silastic tubing. To create the tubing “J” loop, 3/16″ (4.76 mm) female Luer ends were connected to a 3.5-cm (if recellularizing) or 5-cm (if decellularizing) segment of 1/8″ (3.175 mm) × 1/16″ (1.5875 mm) Silastic (Masterflex, Vernon Hills, IL, USA) tube. This piece acted as a nonkinking support that could maintain some stretch in the trachea. A polypropylene circular stage was cut from the end of a 25-mm BD syringe, and two 5/8″ (15.875 mm) holes were drilled to accommodate the Luer ends. A U-shaped loop was made from a 5-cm segment of 2 × 1 mm Silastic tubing with 1/8″ (3.175 mm) male Luer ends. Lastly, the cuffs to which the trachea was connected were 1/8″ (3.175 mm) female Luer ends (if using female tracheas) or 3/16″ (4.76 mm) female Luer ends (if using male tracheas) with barbs truncated to maximize the amount of tissue exposed to the detergent or media. Tracheas were secured to the barbs with 2−0 silk to ensure exclusion of the lumen from the external environment.

**Figure 2:**
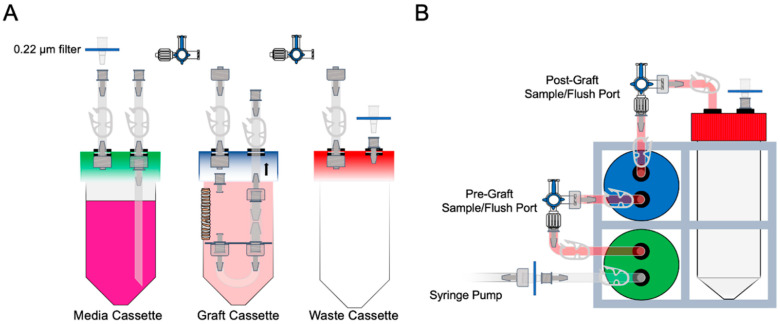
Recellularization. (**A**) Two cassettes, in addition to the bioreactor, were used for the recellularization process: a media cassette and a waste cassette. (**B**) The media cassette was submerged into expansion media, as described, and the waste cassette was fitted onto an empty conical tube. A syringe pump was attached to the 0.22-µm filter and pneumatically forced the media through the tubing at a rate of 0.5 mL/h.

As depicted in Figure 2A, two additional cassettes were required for the recellularization process: a media cassette and a waste cassette. Both caps were fashioned in the same manner in terms of hole sizes and grommets. However, the media cassette had two 1/8″ (3.175 mm) female Luer ends on the outside and a 14-cm 2 × 1 mm Silastic media tube that was connected by a 1/8″ (3.175 mm) female Luer end to the underside of the cap. The waste cap had one 5-cm segment of Silastic tubing with a male Luer end on the outside. The second grommet had one female Luer end directly inserted into it from the outside. Acetal tubing clamps were positioned as depicted (Gizhome Adjustable Mini Flow Control Tube Clamps, UPC 781573726701). A 0.22-µm syringe filter (Thermo Scientific, Waltham, MA, USA) was fitted on the female Luer ends as shown in the figure. This ensured a sterile circuit. Prior to use, the tube caps and external tubing components were sterilized in 70% ethanol for 24 h then rinsed in diH_2_O. The detachable Silastic tubing (“J” loop) and the polypropylene components were autoclaved prior to use.

### 2.4. Decellularization Protocol

Fresh full-length tracheas were procured from ferrets using a sterile technique and stored for 24 h in the previously described antibiotic solution at 4 °C. After decontamination, all adventitial tissue was stripped, and the trachea was sectioned into 5-cm segments and fixed to a graft cassette. This circuit was then submerged into a 50-mL conical tube with 40 mL of DMEM:F12 media with 10% fetal bovine serum (Gibco, Cat#12634028, Grand Island, NY, USA).

**Figure 3:**
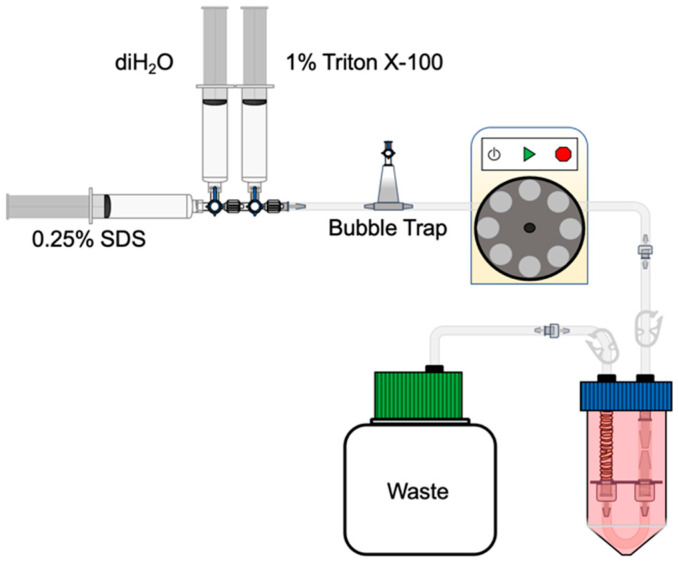
Decellularization. The bioreactor was connected in series to a peristaltic pump and the waste chamber. A protocolized regimen of detergents was perfused through the lumen of the trachea as follows: 0.25% SDS, diH_2_O, and 1% Triton X-100 at 5 mL/min for 8 min each at 37 °C. The trachea was then luminally washed with 1X DPBS at 5 mL/min for 50 min at 37 °C, followed by a 48-h wash at 4 °C.

The Silastic tubing (2 × 1 mm) was connected as shown in Figure 3, and a peristaltic pump was programmed to the following protocol: 10 min with 1X DPBS (100 mL), 8 min with 0.25% sodium dodecyl sulfate (SDS) (*w*/*v*) in deionized water (diH_2_O) (40 mL), 8 min with diH_2_O (40 mL), 8 min with 1% Triton X-100 *v*/*v* in diH_2_O (40 mL), and 50 min with 1X DPBS (250 mL), all at 37 °C. An additional 48 h of cycling with a 1X DPBS wash was performed at 4 °C. Prior to use for recellularization, the tracheas were detached from the circuit by transecting them flush with the cuffs. This ensured the minimal presence of tissue that had not been decellularized in future experiments. The resulting length was approximately 3 cm. The grafts were decontaminated and stored in an antibiotic solution until use.

### 2.5. Recellularization Protocol

For recellularization, a modified perfusion bioreactor was used, as depicted in Figure 2B. Ferret SAEs or murine MECs were expanded until 80−90% confluency and collected with Accutase cell detachment solution (StemCell Technologies, Vancouver, BC, Canada). After the 3-cm decellularized trachea was attached to the graft cassette, the cell pellet was resuspended in 350 µL of either SAGM (for MECs) or Pneumacult Ex Plus (for SAEs). Approximately 2 × 10^6^ cells/cm^2^ luminal surface was required for recellularization. The cell suspension was then slowly injected through the female Luer end until the bolus was observed to reside within the trachea. The tubing clamps were then closed to lock the bolus in place, and the circuit was submerged into an “external” medium consisting of DMEM:F12, 10% (*v*/*v*) FBS, and 1% (*v*/*v*) penicillin/streptomycin. The graft cassette was then mounted on a 1 revolution/minute tube roller for 4 h at 37 °C and then subsequently assembled with the media and waste cassettes for perfusion, as shown in Figure 2A. All tubing clamps were opened, and 10 degrees of angulation to the tubing rack were required to promote the ascension of air bubbles into the waste chamber. The appropriately corresponding expansion media was then pneumatically perfused with a syringe pump at 0.5 mL/min for the duration of the study. The expansion medium was replaced daily, and the “external” medium was replaced every 48 h. Prior to exchanging the perfusion media, 10 mL of 1X DPBS (Gibco, Cat# 14190144, Grand Island, NY, USA) was flushed via a syringe through the pre-graft stopcock to flush nonadherent debris towards the waste chamber.

### 2.6. Establishing an Air–Liquid Interface within the Bioreactor

**Figure 4:**
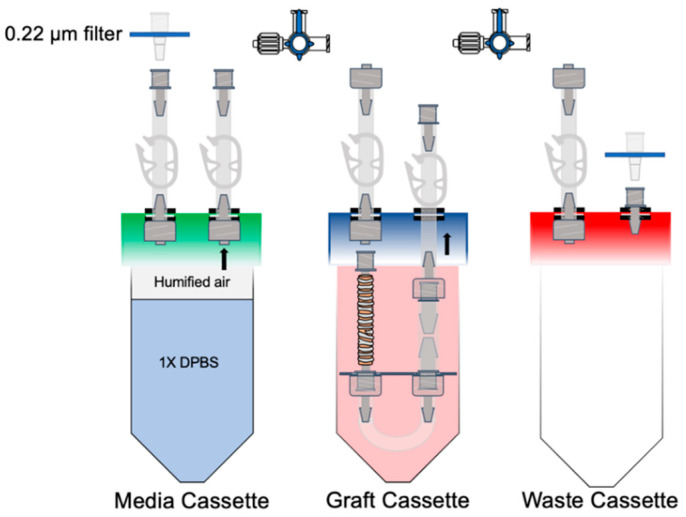
Air–liquid interface set-up. The same cassettes from the recellularization process were used. The media cassette was partly filled with 1X DPBS, and the syringe pump introduced air through the 0.22-µm filter to create humidified air. This was pushed through the circuit at 0.5 mL/h. The circuit was flushed with 5 mL of 1X DBPS daily.

After the tracheal graft had been recellularized and luminally perfused with expansion media for 4 days, the graft was flushed with 1X DPBS to clear debris. The medium in the chamber was replaced with 1X DPBS, and the 14-cm segment of Silastic tubing was removed (Figure 4). The syringe pump continued to deliver humidified air through the graft at a rate of 0.5 mL/hour. The graft was flushed daily as described above.

### 2.7. Live Viability Assay

Normal and decellularized tracheas were rinsed in 1X PBS and then sectioned longitudinally into 0.5-mm sections. Tissue strips were submerged into 0.2 µM of Calcein-AM (Invitrogen, Cat#C1430, Carlsbad, CA, USA) for 25 min, then washed twice in 1X PBS for 5 min. Sections were placed on slides and imaged on a Zeiss 880 (Zeiss, Oberkochen, Germany) confocal microscope.

### 2.8. Compliance Testing

Segments (5 cm) of normal and decellularized tracheas were occluded on one end with a plugged Luer adapter. The other end was attached to a 3-way stopcock with a water column. A syringe injected and aspirated air several times to obviate the effects of hysteresis prior to recording the experiment. Change in pressure within the trachea were measured with repeated injections or aspirations of set volumes of air to calculate tracheal compliance (∆pressure/∆volume).

### 2.9. DNA Quantification

Treated and untreated tracheas were lyophilized for 3 h prior to use. DNA extraction was achieved by means of a DNeasy Blood and Tissue Kit (Qiagen, Germantown, MA, USA), and quantification was performed with Nanodrop.

### 2.10. Immunofluorescence and Histology

Tissues were fixed in 4% paraformaldehyde (PFA) (Sigma-Aldrich, Cat# 158127, St. Louis, MO, USA) at room temperature for 60 min. Tissues were then trimmed to fit a cassette for OCT (Tissue-Tek O.C.T., Sakura, Torrance, CA, USA) embedding in liquid N_2_. Tissues were sectioned at 10 µm and fixed to slides in 4% PFA for 45 min. Antigen retrieval was performed by submersion in a sodium citrate buffer (pH 6.0) overnight at 55 °C. Blocking and permeabilization were conducted with donkey serum and 10% Triton X-100. Primary antibodies and secondary antibodies were incubated for 24 h sequentially. Hematoxylin and eosin staining were used to assess the general tissue architecture. Alcian Blue detected mucins, and Trichrome Masson staining was used to detect collagen.

Whole-mount staining was performed by whole-tissue submersion in 4% PFA at room temperature for 60 min. Antigen retrieval was conducted with a sodium citrate buffer as above with agitation. Blocking and permeabilization required whole-tissue submersion in donkey serum and 10% Triton X-100 for 24 h. Primary and secondary antibodies were sequentially incubated for 24 h each at 37 °C. The tissue was then sandwiched and flattened between two slides and imaged by confocal microscopy.

## 3. Results

### 3.1. Tracheal De-Epithelialization and Assessment of Cellular Presence

**Figure 5:**
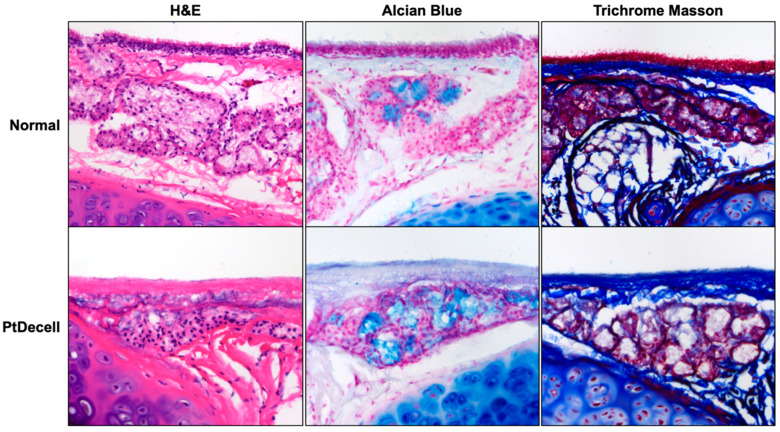
Histologic evaluation of partly decellularized tracheas. Compared with normal tracheas, the partly decellularized tracheas demonstrated the complete absence of luminal cells. Alcian blue staining confirmed that the mucin-containing submucosal glands were still intact and present in the submucosa. Trichrome Masson staining is a comparative stain for collagen, which subjectively appeared to be unchanged on the luminal surface.

**Figure 6:**
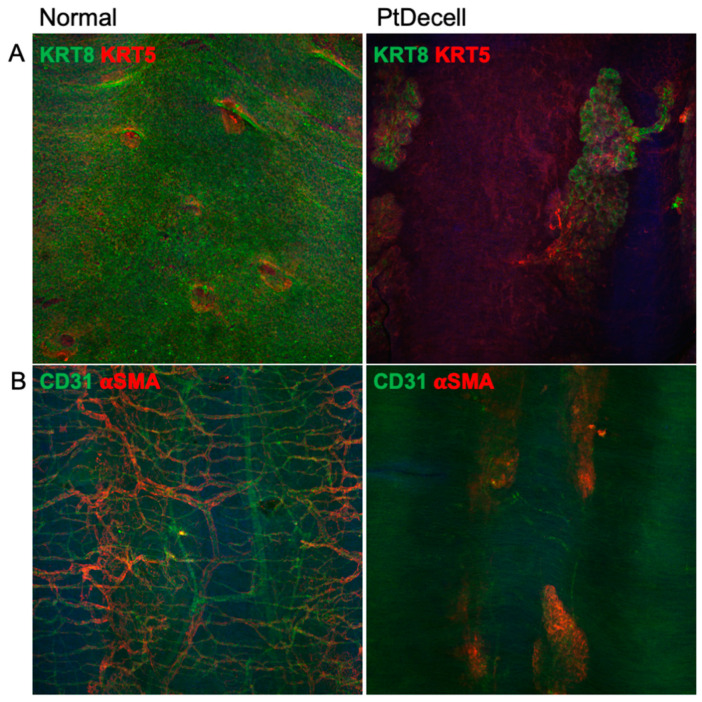
Whole-mount staining of partly decellularized tracheas. (**A**) Keratin 8 and Keratin 5 are markers for luminally committed differentiated cells and basal cells, respectively. Notably, the partly decellularized trachea had no “lawn” of surface airway cells, with the exception of those in the submucosal glands and ducts. (**B**) CD31 is an endothelial cell marker, and α-smooth muscle actin identifies muscular arteries and glandular myoepithelial cells. The decellularized trachea showed an absence of vascularity after treatment.

Histological assessment of the native trachea demonstrated ciliated epithelium and mucin on the luminal surface (Figure 5). In contrast, the corresponding images of the decellularized tracheas demonstrated the complete absence of cells above the basement membrane with retention of the cellular architecture and mucin in the SMGs (Figure 5). Whole-mount staining for keratin (KRT) 5 (a basal cell marker) and KRT8 (a luminal cell marker) were universal on the native tracheal lumen and gland, whereas the decellularized trachea was devoid of surface cells and only exhibited these markers in the SMGs. Similarly, whole-mount staining for vascular markers CD31 (endothelial cells) and αSMA (smooth muscle cells) showed that the treated tracheas had also become largely devascularized (Figure 6).

**Figure 7:**
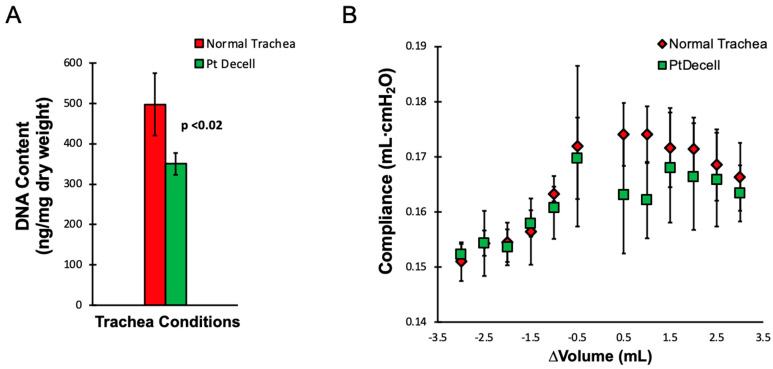
DNA quantification and mechanical analysis. (**A**) DNA quantification between the normal and partly decellularized tracheas showed a significant difference (*p* < 0.05), suggesting that the bulk of DNA and antigenic burden was in the luminal compartment. (**B**) A comparison of tracheal compliance between normal and decellularized conditions showed no difference (*p* > 0.05) across a range of inflated and aspirated volumes. This demonstrates that the partly decellularized tracheas retained their biomechanical integrity.

DNA quantification of the tracheas showed a significant decrease in residual DNA between native and treated tracheas (*p* < 0.02 (Figure 7A). This suggests that the bulk of DNA in tracheas were the nonepithelial cells.

### 3.2. Mechanical Analysis

The compliance of the trachea was contrasted between the native and the treated tissue to ensure that the decellularized trachea maintained its structural integrity. The compliance, measured every 0.5 mL, showed no significant difference between the conditions during inflation and deflation (Figure 7B).

### 3.3. Chondrocyte Viability

Chondrocyte viability is directly correlated with structural integrity, as chondrocytes are responsible for maintenance of the tracheal cartilage. Viability was assessed following the decellularization and washing process through cross-sectional Calcein-AM analysis. The abundance of viable cells was determined by the presence of green fluorescent staining, which is indicative of the intracellular esterase activity needed to process the Calcein-AM.

**Figure 8:**
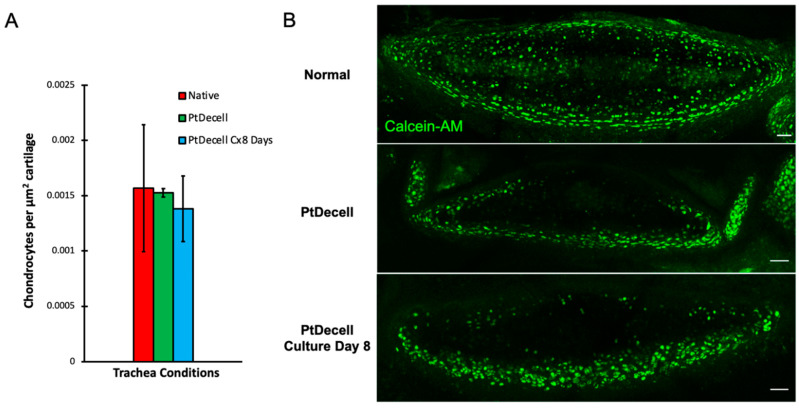
Cartilage viability as determined by a Calcein-AM assay. (**A**) Chondrocyte quantification per unit area of cartilage showed no significant difference between the native and partly decellularized condition (*p* > 0.5) and no significant difference between decellularized tracheas and those cultured for 8 days (*p* > 0.45). (**B**) In the cross-section, fluorescence is a marker of Calcein-AM metabolism and thus viability. There was notable decrease in viability on the luminal rim of the chondrocytes in the partly decellularized and 8-day culture conditions. This is consistent with prior studies, suggesting cell death in portions of the trachea in closest proximity to the cytotoxic detergents. Scale bar: 50 µm.

Native tracheal cartilage showed fluorescence peripherally and centrally, whereas decellularized cartilage had an obvious absence of fluorescence along the periphery adjacent to the lumen. The absence of fluorescence was assumed to suggest cell death. This is consistent with the findings from porcine tracheal de-epithelialization [16], and it suggests that our bioreactor model also preserved the majority of ferret tracheal chondrocytes. Quantification of the viable cartilage occupying the cross-sectional area of the cartilage showed that there was no significant difference in chondrocytes per unit of area (Figure 8A).

Tracheal grafts that were subsequently recellularized with ferret airway basal cells for 8 days in vitro also showed comparable viability in the Calcein-AM assay. This suggests that the extensive 1X DPBS washing period after decellularization was sufficient to prevent residual SDS from causing continued chondrocyte death, and that the bioreactor was capable of maintaining chondrocyte health (Figure 8B).

### 3.4. Recellularization of Decellularized Grafts

Cells chosen for recellularization of decellularized grafts were isolated from animal tracheas and expanded in vitro. Ferret surface airway basal cells (FeSAE) were obtained from a TdTomato-positive ferret and expanded with Pneumacult Ex Plus [33]. Cells were grown in chamber slides and confirmed to have basal cell markers (KRT5/∆Np63/KRT14) [36]. Murine myoepithelial cells (MuMEC) were first induced in vivo by peritoneal injection with tamoxifen, then isolated and expanded with small airway growth media (SAGM) in vitro. Fluorescence-activated cell sorting (FACS) was used to obtain a nearly pure population of GFP-positive cells. MuMECs also exhibited universal KRT5/∆Np63/KRT14 markers, suggesting that they had adopted a basal cell phenotype. None of the mouse cells retained the characteristic α-smooth muscle actin signature.

Both cell candidates were separately engrafted according to the protocol described above. The success of engraftment was determined by the presence of fluorescent cells under immunofluorescence. Recellularization efficiency was assessed at 2 days and 8 days. After the first 4 h of engraftment on the tube roller, the graft underwent the perfusion process. An initial flush of the expansion medium collected the majority of un-engrafted cells in the waste chamber. A cell count in the initial waste medium showed an engraftment efficiency of approximately 90%, regardless of the cell type (data not shown).

**Figure 9:**
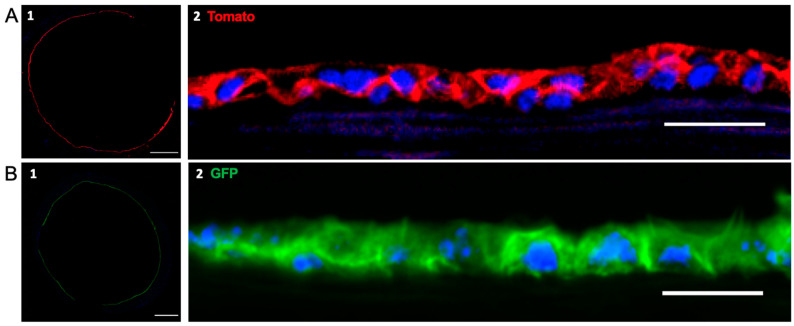
Ferret surface airway epithelium (FeSAE) and murine myoepithelial cell (MuMEC) recellularization on Day 2. (**A1**,**B1**) FeSAEs are marked with Tomato and MuMECs are marked with GFP. After 2 days of recellularization, there was near circumferential seeding of the luminal surface. The trachea was cut to release tension during tissue processing. Scale bar: 1000 µm. (**A2**,**B2**) Longitudinal cross-sections demonstrated a uniform monolayer of cells at 2 days. Scale bar: 25 µm.

On Day 2, both MuMEC and FeSAE grafts showed a uniform and circumferential monolayer on the luminal surface, but there was no engraftment within the submucosa at this timepoint (Figure 9A,B).

**Figure 10:**
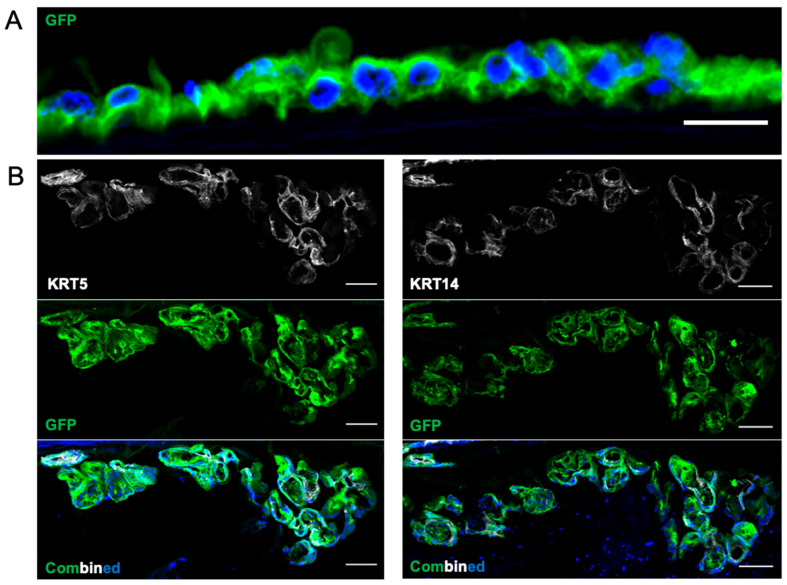
Murine myoepithelial cell recellularization on Day 8. (**A**) Surface MuMECs continued to remain mostly in monolayer without any morphologic change representing differentiated cells. (**B**) Submucosal glands were completely colonized by GFP-positive MuMECs with retention of the basal cell markers: KRT5 and KRT14. There was no differentiation into mucous or serous cells nor reversion into α-smooth muscle actin-positive MuMECs.

On Day 8, both cell populations showed layering on the luminal surface without a morphology resembling differentiated cells. However, whereas the FeSAE grafts had sporadic engraftment within the SMG (not shown), the MuMEC grafts demonstrated complete colonization and tubularization of the SMG resembling those of a native SMG (Figure 10A,B). Immunofluorescent staining showed that all cells still retained basal cell markers, but there was no reversion to αSMA-positive MECs. There were also no markers, such as mucin 5B or lysozyme, that were suggestive of differentiation into glandular mucous or serous cells, respectively. All SMG structures appeared to be in the normal anatomic position between intercartilaginous rings and did not seem to have been generated de novo.

**Figure 11:**
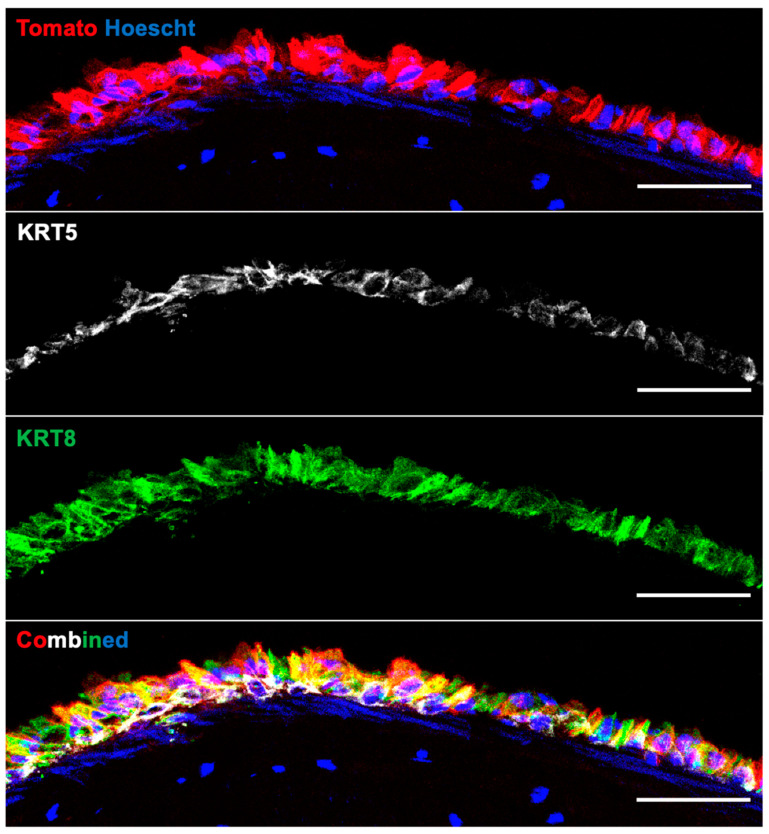
Ferret surface airway epithelium (FeSAE) cultured with an air–liquid interface in the bioreactor on Day 12. Tomato cells were expanded for 4 days and then cultured on an air–liquid interface by syringe-pumping humidified air. There was a clear distinction between cells that retained a Keratin 5 basal cell identity and those that expressed Keratin 8, which indicates commitment to a luminally differentiated cell. There was no expression of cilia or mucin at this early time point.

A subset of experiments using FeSAE attempted to create an air–liquid interface within the bioreactor. Cells were expanded for 4 days with Pneumacult-Ex Plus and then perfused with humidified air for 8 days. Grafts cultured under these conditions were more prone to contamination; however, two successful experiments showed the development of columnar cells with a clear distinction between the KRT5 and KRT8 populations. There was no evidence of ciliated or mucin-producing cells (Figure 11).

## 4. Discussion

Here, we present a reliable and cost-effective bioreactor that is capable of supporting the decellularization and recellularization of ferret tracheas for application in studies of tracheal transplantation that may become an important tool for studying stem cell engraftment and differentiation. With exception of the need for a peristaltic pump and syringe pump, the entire bioreactor can be mass-recreated with a minimal budget. The design was inspired by work from Loy et al. [35], and the detergent-based protocol was adapted for ferrets from Aoki et al. [16]. Ferrets are clinically advantageous for the study of tracheal disease for several reasons: (1) the cost of maintenance per animal is considerably less than that of larger animals (e.g., pigs); (2) their tracheas are generously long, equating to more experiments per sacrificed animal; (3) their airways—like those of humans—have SMGs throughout the entire cartilaginous airways, unlike rodents; and (4) their histologic profiles of chronic rejection in airway transplantation experiments are identical to those in humans [28,29,30]. In anticipation of orthotopic transplantation experiments, ferrets are also amenable to bronchoscopic examination.

As stated in prior studies, the benefits of exclusive decellularization of the lumen are the considerably shorter processing time and the preservation of tracheal cartilage. There are numerous decellularization protocols, e.g., detergent-based, which require hours to days of decellularization before experimental use [7,37,38,39]. The advantage of Aoki’s porcine partial de-epithelialization protocol is a quicker turnaround of 4 h and 40 min, and this becomes even more apparent in the smaller ferret trachea (74 min).

The preservation of tracheal cartilage is also clinically important and should not be understated. Currently, the process of cartilage regeneration in decellularized grafts remains a challenge due to the inadequate penetration of stem cells into the cartilage lacunae [40]. With our partial graft decellularization, we obviated the need for cartilage engraftment. Prior studies conducted by Liu et al. and Lu et al. showed that maintaining cartilage viability in a canine tracheal graft was necessary for preventing eventual stenosis and maintaining airway patency [15,41]. They specifically noted that the retention of approximately 50% of tracheal chondrocytes was sufficient to prevent the development of lethal stenosis in a 4-week orthotopic transplantation study. Decellularized ferret tracheas demonstrated that tracheal chondrocytes were generally viable after processing, with a notable absence of viability in the areas nearest to detergent perfusion. The quantity of chondrocytes per unit of cartilage changed insignificantly after in vitro culturing for 8 days. Differences in compliance were also insignificant between native and freshly treated tracheas, suggesting the maintenance of structural integrity and that the tracheas will be less likely to collapse if transplanted in vivo. Additionally, chondrocytes are immune-privileged, and preservation of native cells would not increase the risk of rejection following transplantation [17].

The act of decellularization is critical to reducing the antigenic burden after transplantation. Epithelial cells are a primary source of MHC mismatch and are thus an important immunologic target during the process of chronic allograft rejection [18,19,42,43,44,45]. Our decellularization protocol showed a complete absence KRT5-positive and KRT8-positive cells on the luminal surface, which is clinically useful for future transplantation. However, SMGs remained intact; these are a source of MHC mismatch, which can trigger CD4+ T cell proliferation and potential graft rejection after transplantation [22]. This is an undesired consequence of partial decellularization in an effort to preserve the tracheal cartilage, and further studies are required to evaluate the fate of residual SMG tissue after recellularization and their contribution to orthotopic transplant.

Recellularization of tracheal grafts have also been shown to be critical in preventing rejection or development of bronchiolitis obliterans [20,21,46,47,48]. Recellularization with recipient-derived stem cells would be practically ideal, but, here, we present data regarding re-epithelialization with allogeneic and xenogeneic cell candidates. Using fluorescent Tomato-positive surface airway basal cells, we demonstrated that our bioreactor was capable of maintaining a three-dimensional tissue culture. We observed a uniform and circumferential coverage of the entire graft. Similarly, GFP-positive mouse MECs expressing basal cell-like features were also able to successfully engraft on the partly decellularized scaffold over 8 days. The most interesting aspect of this subset of experiments is the complete colonization of the native SMGs, suggesting that MECs have a strong propensity to engraft the SMGs, consistent with their ability to repair gland structures in the face of severe injury [25]. The success of MEC engraftment within the SMG may be due to the gradual loss of endogenous cells from ischemia. When the trachea is harvested and devascularized, SMGs lose their source of nutrition and oxygen via capillary delivery from the adventitial layer. Conversely, MECs from recellularization are fed from luminal expansion media, and thus have a competitive advantage to reconstruct the SMGs. In the same vein, there was no evidence of MEC differentiation into functional SMG cells—such as mucous or serous cells—which we attributed to the expansion media, which was designed to preserve the stem cells’ properties.

Lastly, a subset of experiments aimed at generating an air–liquid interface within the bioreactor was performed. This idea stemmed from the experiments of Kobayashi et al., wherein gingival fibroblasts co-cultured with tracheal epithelial cells produced a functionally differentiated airway epithelium [49]. Through our method of partly decellularizing the trachea, we preserved not only the tracheal cartilage but also the supportive fibroblasts as well. Our theory was that the surviving fibroblasts would be able to provide signals that induced the differentiation of SAE basal cells. By perfusing the lumen with air and creating an adventitia-to-lumen nutrient gradient, we established a culture paradigm similar to the Transwell air–liquid interface protocols used for differentiating airway stem cells [50]. In an experimental setup of 4 days of expansion followed by 8 days of propagation with humidified air, we were able to—albeit inconsistently—generate an immature epithelium that showed distinct KRT8 expression within our engrafted cell population. KRT8 is a marker of a luminal lineage’s commitment to ciliated or mucus-producing cells, and this proof-of-concept study could potentially generate a fully functional tracheal graft in vitro prior to transplantation [51]. Through refining this technique, it is possible that establishing an air–liquid interface may also induce functional differentiation of the MECs colonized within the SMG.

Our study is not without limitations. Chief among these is fact that native SMG remnants were evident through the decellularized graft. This is consistent with the porcine grafts presented by Aoki et al. An ideal graft would be purged of any donor antigens, but this is an acceptable trade-off for maintaining viable cartilage. Our study was able to show that murine MECs could completely occupy the SMGs, but we did not establish if there were residual cells from the native trachea. Another limitation is that the bioreactor is only capable of accommodating female tracheas. The caliber of male tracheas is too wide for the 1/8″ (3.175 mm) Luer barb in the bioreactor, which leads to inconsistent decellularization. This may be modified by using a 3/16″ (4.76 mm) Luer barb instead, but this limits the usable length of the trachea. Lastly, we have been unable to consistently establish a sterile air–liquid interface due to early contamination once the medium with suppressive antimicrobials was discontinued, and we only had two samples with successful differentiation.

In summary, this study introduced a trachea bioreactor with the following advantages over existing models: reduced duration of tissue-processing, retention of graft biomechanics, and a possibility for in vitro SMG and SAE differentiation in an anatomically correct model. Future orthotopic transplantation of our grafts in ferrets can provide clinically translatable information for human application.

## Data Availability

Not applicable.
